# Wnt3a: functions and implications in cancer

**DOI:** 10.1186/s40880-015-0052-4

**Published:** 2015-09-14

**Authors:** Sha He, Yi Lu, Xia Liu, Xin Huang, Evan T. Keller, Chao-Nan Qian, Jian Zhang

**Affiliations:** Key Laboratory of Longevity and Ageing-related Diseases, Ministry of Education, Nanning, Guangxi 530021 P.R. China; Center for Translational Medicine, Guangxi Medical University, Nanning, Guangxi 530021 P.R. China; Department of Urology and Pathology, School of Medicine, University of Michigan, Ann Arbor, MI 48109 USA; Department of Nasopharyngeal Carcinoma, Sun Yat-sen University Cancer Center, State Key Laboratory of Oncology in South China, Collaborative Innovation Center of Cancer Medicine, Guangzhou, Guangdong 51006 P.R. China

**Keywords:** Wnt3a, The Wnt signaling pathway, Cancer

## Abstract

Wnt3a, one of Wnt family members, plays key roles in regulating pleiotropic cellular functions, including self-renewal, proliferation, differentiation, and motility. Accumulating evidence has suggested that Wnt3a promotes or suppresses tumor progression via the canonical Wnt signaling pathway depending on cancer type. In addition, the roles of Wnt3a signaling can be inhibited by multiple proteins or chemicals. Herein, we summarize the latest findings on Wnt3a as an important therapeutic target in cancer.

## Background: the Wnt signaling pathway

The *Wnt* gene was first identified in 1982 and is named the *Int* gene in mice [1]. A subsequent study reported that the *Int* gene and wingless gene in *Drosophila* were homologous genes [2]. Therefore, both genes were recognized as the *Wnt* gene [3]. The Wnt family comprises 19 human proteins, including Wnt1, Wnt2, Wnt2b (Wnt13), Wnt3, Wnt3a, Wnt4, Wnt5a, Wnt5b, Wnt6, Wnt7a, Wnt7b, Wnt8a, Wnt8b, Wnt9a (Wnt14), Wnt9b (Wnt14b), Wnt10a, Wnt10b, Wnt11, and Wnt16. These genes encode secreted glycoproteins that are rich in cysteine [4]. Wnts can combine with cell membrane receptors that play a critical role in autocrine regulation and/or participate in paracrine modification by binding to adjacent cell membrane receptors. The signal transduction pathway mediated by *Wnt* genes is called the Wnt signaling pathway.

**Fig. 1:**
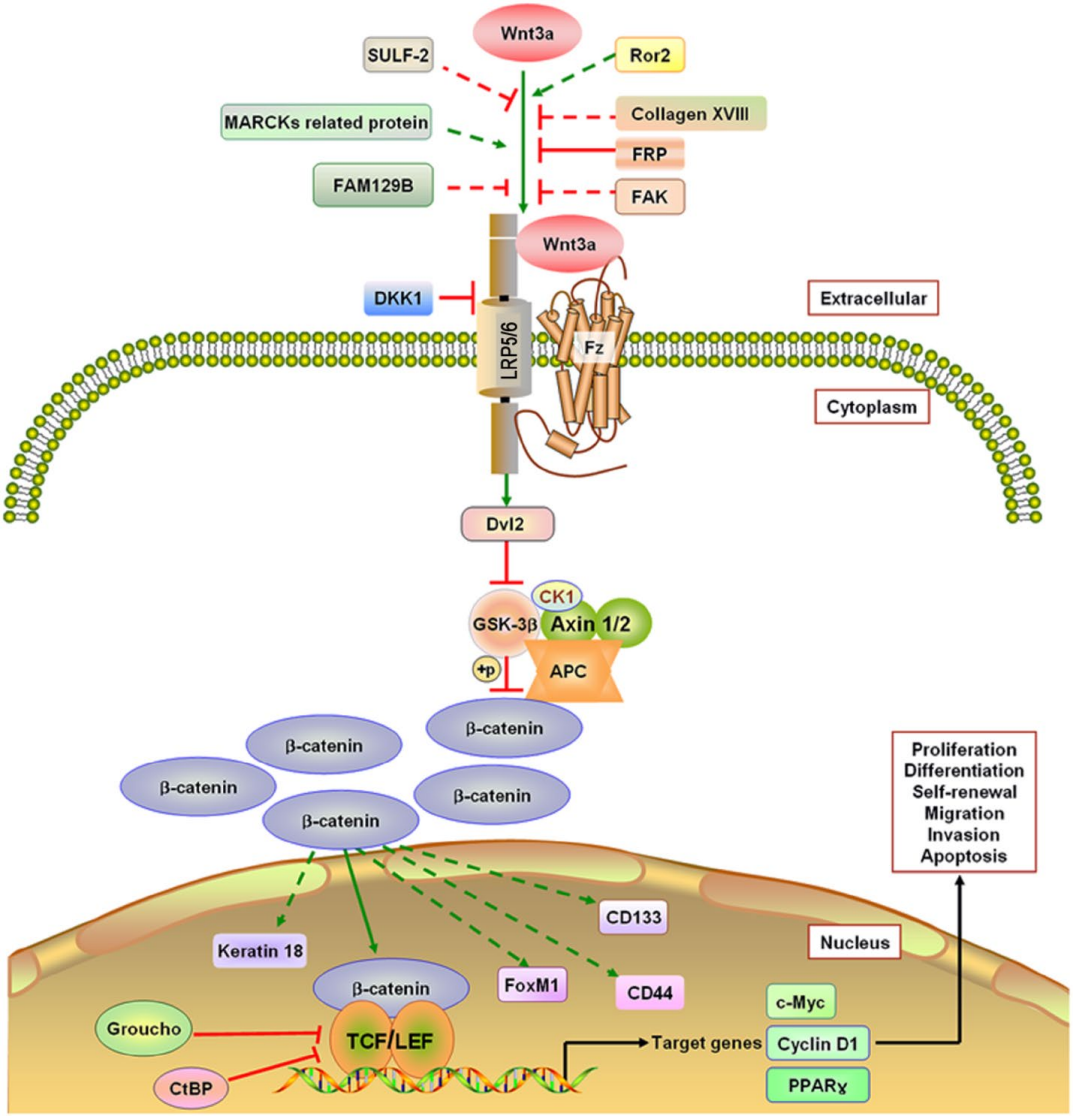
The canonical Wnt3a pathway. *SULF-2* sulfatase 2, *Ror2* an orphan receptor, belonging to the Ror family of receptor tyrosine kinases, *MARCKs* myristoylated alanine-rich C-kinase substrate, *FAM129B* family with sequence similarity 129, member B, *FAK* focal adhesion kinase, *DKK1* Dickkopf-related protein 1, *LRP5/6* lipoprotein receptor-related protein 5/6, *Dvl* disheveled, *GSK-3β* glycogen synthase kinase-3β, *CK1* casein kinase 1, *CtBP* C-terminal binding protein, *PPARγ* peroxisome proliferator-activated receptor-gamma, *TCF* T-cell–specific transcription factor, *LEF* lymphoid enhancer-binding factor, *FRP* frizzled-related protein, *Axin1/2* axis inhibition protein 1 and 2, *APC* adenomatous polyposis coli, *FoxM1* forkhead box M1

The Wnt signaling pathway can be divided into canonical and noncanonical pathways. The canonical pathway is also called the Wnt/β-catenin pathway (Fig. 1). The noncanonical pathway includes the Wnt–planar cell polarity pathway (Wnt-PCP pathway) and the Wnt-calcium pathway (Wnt-Ca^2+^ pathway) [5]. According to the characteristics of its functions, the Wnt family can also be divided into two categories: Wnt1 and Wnt5a. The Wnt1 category includes Wnt1, Wnt2, Wnt2b, Wnt3, Wnt3a, Wnt7a, Wnt8, Wnt8b, and Wnt10a, which are involved in the canonical signaling pathway, whereas Wnt4, Wnt5a, and Wnt11 belong to the Wnt5a category and activate the noncanonical signaling pathway.

The canonical Wnt pathway begins with the binding of a Wnt ligand to its receptor Frizzled (Fz) and co-receptor lipoprotein receptor-related protein (LRP5/6), which then activates the scaffold protein Disheveled (Dvl) and causes dissociation of the “destruction complex” formed with casein kinase 1 (CK1), glycogen synthase kinase-3β (GSK-3β), and the scaffolding proteins adenomatous polyposis coli (APC), Axin1, and Axin2 [6, 7]. The dissociation of the “destruction complex” causes degradation of β-catenin, therefore inducing the cytoplasmic accumulation of β-catenin and its translocation into the nucleus to interact with T cell–specific transcription factor (TCF) and lymphoid enhancer-binding factor (LEF), which regulate the expression of Wnt target genes [6, 7]. The Wnt/β-catenin signaling pathway is one of the most conserved pathways in organism evolution and plays key roles in embryonic development, cell growth, differentiation, polarity formation, neural development, and carcinogenesis [8]. Aberrant activation of the Wnt/β-catenin signaling pathway has been implicated in a wide variety of cancer types [9, 10], and β-catenin has been demonstrated to be a crucial factor for regulation of Wnt/β-catenin downstream target genes [9]. Nuclear accumulation of β-catenin has been recognized as a biomarker for cancer prognosis [11, 12].

## Wnt3a in non-cancer cell development

The *Wnt3a* gene, clustered on human chromosome 1q42 [13], induces the accumulation of β-catenin, thereby activating the canonical Wnt signaling pathway [14]. It plays crucial roles in both proliferation and differentiation processes in several types of stem cells, such as neural stem cells and hematopoietic stem cells. In addition, Wnt3a can stimulate the migration and invasion of trophoblasts [15] and induce the survival, proliferation, and migration of human embryonic kidney (HEK) 293 cells [16]. Wnt3a has also been shown to induce the migration, invasion, and colony formation of mouse mammary epithelial HC11 cells [17]. In addition, Wnt3a can down-regulate E-cadherin expression and increase the proliferative potency of ovarian surface epithelium (OSE) cells [18]. Wnt3a promotes cell survival and the formation of spheroids in colonic epithelial cells or crypts [19] and inhibits apoptosis in mouse embryonic liver stem cells, prolonging their survival by up-regulating anti-apoptotic factors and down-regulating pro-apoptotic factors of the Bcl-2 family [20]. Wnt3a up-regulates genes implicated in melanocyte differentiation [21] and increases the expression and nuclear localization of the transcriptional co-activator with PDZ-binding motif (TAZ), a transcriptional modulator that plays a key role in cell proliferation, differentiation, and stem cell self-renewal, activating osteoblastic differentiation [22]. This process also induces osteoblastic differentiation and the expression of osteoprotegerin (OPG) in osteoblast precursor C2C12 cells [23]. Furthermore, Wnt3a inhibits B lymphopoiesis and plasmacytoid dendritic cells but promotes the retention of hematopoietic stem cell markers, cell yields [24], and the self-renewal of hematopoietic stem cells [25]. Wnt3a suppresses the differentiation of mesenchymal stem cells into osteogenic or adipogenic lineages and Wnt3a-producing TW3R feeder cells, providing a growth advantage for undifferentiated human induced pluripotent stem cells (iPSC) and embryonic stem cells (ESCs) [26]. Wnt3a promotes the proliferation of human ESCs [27]. Wnt3a induces cell proliferation, morphological changes, and migration in C18-4 spermatogonial stem cells, and it maintains adult human embryonic stem (ES)-like cells derived from human germ cells in an undifferentiated stage, expressing essential human ES cell transcription factors [28]. Co-culture of Wnt3a-producing rat embryonic fibroblasts with human pre-B leukemia cell lines KM3 and REH significantly reduces Apo2L/TNF-related apoptosis-inducing ligand (TRAIL)-induced apoptosis of the lymphoid cells [29]. Wnt3a expression leads to stabilization and nuclear accumulation of β-catenin in primary lineage-restricted B progenitor cells, suppresses B lymphopoiesis of CD133^+^CD10^−^ hematopoietic progenitor cells and CD10^+^ B progenitor cells, and suppresses cell division of B progenitor cells [30]. Wnt3a promotes c-Kit^−^ cells to induce myeloid and erythroid progenitors with robust self-renewal capacity. Wnt3a-stimulated c-Kit^−^ cells induce all hematopoietic lineages (lymphoid, myeloid, and erythroid) upon transplantation into the liver of newborn recipient mice [31].

Wnt3a also plays important roles in organ formation. It can enhance cardiac myogenesis by increasing TCF-dependent transcription and inducing cardiac-specific markers, bone morphogenetic proteins (BMPs), and sarcomeric myosin heavy chains (MHC) [32]. Wnt3a has been shown to induce myogenesis in the myotome of the differentiating somite and the activation of myogenic differentiation antigen (MyoD) expression and subsequent skeletal muscle development [33].

Wnt3a actives atypical protein kinase C ioys (pKCiota) and induces a punctate distribution of pKCiota in the neurites and cytoplasm, with a particularly intense signal at the centrosome [34]. Wnt3a serves as a crucial player in neuro-mesodermal stem cell maintenance and differentiation [35]. Therefore, Wnt3a is involved in the formation of the nervous system.

It has been reported that Wnt3a induces production of bone-protective OPG and alkaline phosphates in preosteoblastic C2C12 cells [36]. It can also induce natural killer T cell anergy to re-stimulate alpha-GalCer [37]. Wnt3a prevents the adverse effects of Lgr4 loss on organoid formation [38] and induces EphB4 in mesenchymal stem cells [39]. Wnt3a increases the percentage of CD34^−^CD7^+^ and CD34^−^CD7^+^cyCD3^+^ cells and the expression of CD3 epsilon and preT alpha [40]. Wnt3a overexpression is positively associated with ulcerative colitis versus noninflammatory bowel disease [41]. Wnt3a induced Fz1 expression in a normal colon mucosa–derived cell line in vitro [42]. It has been demonstrated that Wnt3a mediates somitogenesis by activating a network of interacting target genes that promote mesodermal fate and position boundary determination genes, thus activating the segmentation clock in the anterior presomitic mesoderm [35].

## Wnt3a expression in tumors

Tumor development depends upon the interactions among environmental factors, cells in the tumor microenvironment, and the genetic nature of tumor cells, leading to the disordered expression of oncogenes, tumor suppressor genes, and abnormal intracellular signal transduction pathways. Studies on intracellular signal transductions have become “hot” research fields in terms of tumorigenesis, invasion, and metastasis. Abnormal activation of the Wnt signaling pathway has been shown to be closely associated with tumor progression and be involved in various types of cancer. For example, Wnt3a is highly expressed in human scirrhous gastric carcinoma 44As3 cells with highly metastatic derivatives [43], advanced prostate cancer cells [44], and activating transcription factor (ATF3)-induced mammary tumors [45]. Wnt3a expression is higher in most breast cancer cell lines than in normal human mammary epithelial cells [46], which is also true in oral squamous cell carcinoma compared with control nonmalignant tissues [47]. In addition, Wnt3a expression is associated with the clinical grade and aggressiveness of gliomas, and interestingly, it is overexpressed on glioma stem cells [48]. *Wnt3a* and *Wnt3* mRNA were reported to be co-expressed in the embryonal carcinoma NT2 cell line and the breast cancer MCF-7 cell line [49].

## Wnt3a suppresses or promotes cancer cell growth in vitro

Wnt3a can either suppress or promote cancer cell growth, depending upon cancer types. For example, Wnt3a suppresses the growth and proliferation of the lung and lacrimal gland [50], induces B-cell precursor acute lymphoblastic leukemia (B-ALL) cell death [51], and suppresses the proliferation of several B-ALL cell lines [52]. Wnt3a enhances TRAIL-dependent apoptosis in multiple melanoma cell lines [53]. Wnt3a decreases proliferation rate and induces myogenic differentiation in alveolar rhabdomyosarcoma cells [54]. On the other hand, Wnt3a antagonizes the inhibition of the self-renewal of liver cancer stem cells induced by 8-bromo-7-methoxychry [55], promotes the self-renewal of leukemic stem/progenitor cells in some acute myeloid leukemia and acute T-lymphoblastic leukemia cell lines [25], promotes the proliferation and survival of acute lymphoblastic leukemia cells [56], reduces the anti-proliferative activity of lenalidomide multiple myeloma [57], and stimulates the proliferation of malignant mesothelioma cells [58]. Moreover, Wnt3a promotes the proliferation of breast cancer MCF7 cells [59] and increases the expression of high-mobility group A protein 1 to maintain the proliferation of gastric cancer cells [60]. Wnt3a reverses docosahexaenoic acid-induced growth inhibition in human pancreatic cancer PANC-1 cells [61]. Increased Wnt3a expression induces prostate cancer LNCaP cell proliferation, neutralizing antibodies to reverse Wnt3a tumorigenesis [62]. Wnt3a can enhance hypoxia-induced epithelial-mesenchymal transition (EMT) in hepatocellular carcinoma (HCC) [63]. Wnt3a may play a key role in the maintenance of NT2 cells in the undifferentiated proliferation stage [49]. When 4T1 cells are treated with recombinant Wnt3a along with quercetin, Wnt3a is able to restore the suppressed cell viability by quercetin [64]. Furthermore, Wnt3a treatment significantly decreases the drug susceptibility of cholangiocarcinoma QBC939 cells to common chemotherapeutics [65], sensitizes malignant mesothelioma cells to cytotoxic drugs [58], and induces striking morphological changes accompanied by the rearrangement of the actin cytoskeleton in myeloma cells [66].

## Wnt3a affects tumor growth in vivo

It has been reported that based on the expression of *Wnt3a*, erb-b2 receptor tyrosine kinase 3 (*ERBB3*), lymphocyte-specific protein tyrosine kinase (*LCK*), and Rho family GTPase 3 (*RND3*) to predict mortality, the four-gene model achieved better predictive value than clinical stage or tumor size in early-stage non–small cell lung cancer [67]. Wnt3a decreases metastases in a mouse melanoma model [68]. However, the Wnt3a transduced into HCC cells may promote cell cycle progression and accelerate tumor formation in athymic nude mice [69]. Co-injecting Wnt3a-expressing human mammary fibroblasts with human breast cancer cell lines into mouse mammary fat pads accelerates tumor growth [70]. Wnt3a treatment abolishes sorafenib-induced HCC growth inhibition in vitro and in vivo [71]. Wnt3a-expressing H929 cells injected into human bone exhibit increased osteoblast-to-osteoclast ratios and therefore reduce tumor burden. Likewise, treatment with recombinant Wnt3a stimulates bone formation and attenuates multiple myeloma cell growth in myelomatous severe combined immunodeficiency (SCID)-hu mice [72].

## Wnt3a functions in cancer

### Wnt3a modulates gene expression in cancer

The apoptosis signal-regulating kinase 1 (ASK1) protein expression level is reduced in L929 cells that stably express Wnt3a versus L929 control cells [73]. Wnt3a causes increased self-renewal and prostasphere size, which is associated with a significant increase in nuclear β-catenin, keratin 18, CD133, and CD44 expression in prostate cancer cells [74]. Wnt3a increases the level of forkhead box M1 (FoxM1), which binds directly to β-catenin, enhancing β-catenin nuclear localization and transcriptional activity in tumor cells [75], and increases prostaglandin E2 (PGE2) levels through very early 15-hydroxyprostaglandin dehydrogenase (15-PGDH) suppression to promote colorectal tumorigenesis [76]. Wnt3a can enhance the expression and activity of phospholipase D1 (PLD1; a cell survival mediator) in many types of cancer cells [77] and enhance the expression of PLDs at a transcriptional level, as shown in colorectal cancer HCT116 cells. PLD is necessary for the Wnt3a-driven invasion and anchorage-independent growth of colon cancer cells [78]. Wnt3a induces BMP-4 and BMP-6 expression and their promoter activation in prostate cancer cells [79]. It significantly increases junction plakoglobin, interacts with sex-determining region Y-related high-mobility group box 4 (SOX4) in both the cytosol and the nucleus, and enhances the interaction between SOX4 and plakoglobin in prostate and breast cancer cells [80]. In prostate cells, Wnt3a can recruit the androgen receptor (AR) to the promoter regions of c-Myc and cyclin D1 [81]. Wnt3a either induces AR activity in the absence of androgen or enhances AR activity in the presence of low concentrations of androgen, and significantly enhances growth of LNCaP cells in the absence of androgen [82]. Wnt3a expression is significantly associated with lymph node involvement and matrix metallopeptidase-9 **(**MMP-9) expression in primary tumor, mesenchyme adjacent to tumor, and metastatic sites in colorectal cancer [83]. It has been reported that the expression of the Wnt pathway genes is associated with estrogen receptor (ER) expression, and specific Wnt genes can predict relapse within specific subtypes of breast cancer. Wnt3a can increase mammosphere formation in ER-positive breast cancer cell lines; however, only ER-negative mammospheres are responsive to the ligand Wnt3a in patient-derived metastatic breast cancer samples [84].

### Wnt3a is regulated by genes/proteins in cancer

Sulfatase 2 (SULF2) increases Wnt3a expression in HCC cells, and xenografts established from SULF2-transfected Hep3B cells show increased Wnt3a levels in nude mice [85]. Dvl2 may increase prostate cancer growth and metastatic potential by up-regulating Wnt3a expression [86]. Ror2 positively modulates the Wnt3a-activated canonical Wnt signaling pathway in lung carcinoma H441 cells [87].

## Potential inhibitors of Wnt3a signaling in preclinical studies

Increasing evidence has demonstrated that a number of proteins and chemicals can inhibit Wnt3a signaling in vitro and in vivo (Table 1).

**Table 1:** Potential inhibitors of Wnt3a

Tissue type	Cell line(s)	Potential inhibitor(s) of Wnt3a	Reference(s)
Breast cancer	EpRas MDA-MB-231, T-47D MDA-MB-231 MCF-7	Knockdown of MARCKS-related protein Niclosamide and silibinin Kallistatin Deep-sea water β-estradiol Knockdown of FAK protein	[88] [111, 112] [99] [114] [49] [95]
Colon cancer	SW480	MEK inhibitor PD98059, genistein, and wortmannin	[100]
Gastric cancer	Gastric cancer cells	Receptor for activated protein kinase C	[103]
Leukemia	U937	Cordycepin	[107]
Liver cancer	Huh-7	Variant 3 of collagen XVIII HS20	[89] [96]
Multiple myeloma	OPM-2, and MM patients’ plasma cells	DKK1	[92]
Melanoma	A375 and A2058	Loss of FAM129B	[94]
Prostate cancer	C4-2B PC-3 and DU145	Axin Niclosamide and silibinin	[79] [111, 112]
Renal carcinoma	ACHN	Enhancer of zeste homolog 2 small interfering RNA	[93]
Teratocarcinoma	F9 NT2	RGS19 All-trans-retinoic acid	[104] [115]
HEK293	HEK293	Etinoic acid Variant 3 of collagen XVIII Purified recombinant frizzled cysteine-rich domain MEK inhibitor PD98059, genistein, and wortmannin Aspirin and norcantharidin Hexachlorophene Demethoxycurcumin and bisdemethoxycurcumin Mesd C-terminal region peptide and full-length Mesd protein A diterpenoid derivative, 15-oxospiramilactone Niclosamide and silibinin Magnolo l	[49] [89] [98] [100] [108] [109] [110] [105] [106] [111, 112] [116]
Embryos	Cytotrophoblasts Neural plate	DKK1 Knockdown of FAK	[15] [95]
B lymphopoiesis	CD133^+^CD10^−^CD10^+^ cells	DKK1	[30]
Fibroblasts	NIH3T3	Chondroitin sulfate-E	[97]
Osteoblasts Osteoblast-like cells	Osteoblasts Saos-2, MG63, C2C12, and hFOB1.19 C2C12	DKK1 DKK1 Mesd C-terminal region peptide	[90] [91, 92] [105]

*MARCKS* myristoylated alanine-rich C-kinase substrate, *FAK* focal adhesion kinase, *MEK* mitogen activated and extracellular regulated kinase, *HS20* a human monoclonal antibody against GPC3, *GPC3* glypican-3, *DKK1* Dickkopf-related protein 1, *FAM129B* family with sequence similarity 129, member B, *RGS19* regulator of G-protein signaling 19

### Proteins

Knockdown of myristoylated alanine-rich C-kinase substrate (MARCKS)-related protein in EpRas mammary epithelial cells reduces Wnt3a-induced TCF reporter signaling in vitro and causes loss of tumorigenesis in vivo [88]. Variant 3 of Collagen XVIII suppresses Wnt3a-induced stabilization of β-catenin and then down-regulates cyclin D1 and c-Myc to reduce tumor cell growth in colorectal and liver cancer cell lines [89]. Dickkopf-related protein 1 (DKK1) inhibits the Wnt3a-regulated OPG and receptor activator of NF-kappaB ligand (RANKL) expression in osteoblasts, indirectly inducing osteoclast function [72, 90, 91]. DKK1 blocks Wnt3a-induced β-catenin accumulation in multiple myeloma [92] and Wnt3a-mediated B lymphopoiesis of CD133^+^CD10^−^ hematopoietic progenitor cells and CD10^+^ B progenitor cells [30]. Pre-treatment with recombinant DKK1 completely abolishes Wnt3a-induced OPG expression in mouse and human osteoblasts. Wnt3a-induced OPG expression could be diminished in osteoblasts co-cultured with a DKK1-expressing multiple myeloma (MM) cell line or primary MM cells. DKK1 blocks Wnt3a-stimulated trophoblast migration and invasion through matrigel and also reduces basal migration, invasion, and proliferation of cytotrophoblasts [15]. The enhancer of zeste homolog 2 small interfering RNA reduces the expression of Wnt3a and β-catenin in renal cell carcinoma and markedly inhibits the proliferation and invasion capabilities of human renal carcinoma ACHN cells [93]. FAM129B is needed for Wnt3a to activate a β-catenin–dependent reporter and decreases the ability of Wnt3a to increase the expression of the β-catenin target gene *Axin2*. Loss of FAM129B inhibited the apoptosis of melanoma cells induced by Wnt3a [94]. Knockdown of focal adhesion kinase (FAK) down-regulates Wnt3a gene expression in the neural plate [95]. HS20, a human monoclonal antibody against glypican-3 (GPC3), blocks Wnt3a/β-catenin signaling. Moreover, HS20 inhibits Wnt3a-dependent cell proliferation in vitro and HCC xenograft growth in nude mice [96]. Chondroitin sulfate-E decreases Wnt3a signaling through the negative regulation of LRP6 receptor activation in NIH3T3 fibroblasts [97]. Purified recombinant frizzled cysteine-rich domain inhibits Wnt3a-induced β-catenin activation in vitro [98]. Kallistatin inhibits the Wnt3a-induced proliferation, migration, and invasion of breast cancer cells. Furthermore, kallistatin suppresses the Wnt3a-mediated phosphorylation of LRP6 and GSK-3β and the elevation of cytosolic β-catenin levels. Kallistatin has been shown to antagonize the expression of Wnt3a-induced target genes, including *c*-*Myc*, *cyclin D1*, and vascular endothelial growth factor (*VEGF*) [99]. The mitogen activated and extracellular regulated kinase (MEK) inhibitor PD98059, genistein, and wortmannin effectively suppress Wnt3a/β-catenin–regulated transcriptional activities as well as the intracellular accumulation of β-catenin protein in human colon cancer cells [100]. Axin blocks Wnt3a’s induction of BMP promoters in prostate cancer C4-2B cells [79]. Small interfering RNA of LRP5/6, secreted frizzled-related protein 2 (sFRP2), or sFRP5 potently reduce Wnt3a-mediated β-catenin accumulation [101]. The protein kinase N1 inhibits Wnt/β-catenin signaling and sensitizes melanoma cells to cell death stimulated by Wnt3a [102]. The receptor for activated protein kinase C inhibits Wnt3a signaling by inhibiting the recruitment of Axin by Dvl2 and by stabilizing the β-catenin destruction complex and acts as a tumor suppressor in gastric cancer cells [103]. Regulator of G-protein signaling 19 (RGS19) specifically reduces Wnt-responsive gene transcription in time-dependent and dose-dependent manners and blocks Wnt3a-induced cytosolic β-catenin accumulation, Dvl3 phosphorylation, and the formation of primitive endoderm [104]. The Mesd C-terminal region peptide and the full-length Mesd protein block Wnt3a-induced Wnt/β-catenin signaling in LRP5- and LRP6-expressing cells, inhibit Wnt/β-catenin signaling in human breast HS578T cells and prostate cancer PC-3 cells, and inhibit cancer cell proliferation [105].

### Chemicals

The diterpenoid derivative 15-oxospiramilactone suppresses Wnt3a-stimulated top-flash reporter activity in HEK293T cells and inhibits the growth of colon cancer SW480 and Caco-2 cells [106]. Cordycepin suppresses the effect of Wnt3a-induced β-catenin in leukemia cells [107]. Norcantharidin reduces 80% of Wnt3a-induced β-catenin binding with lymphoid enhancer factor/T-cell factor protein [108]. Hexachlorophene antagonizes the Wnt3a-induced β-catenin response transcription (CRT) by promoting the degradation of β-catenin. In addition, hexachlorophene represses the expression of cyclin D1 and inhibits the growth of colon cancer cells [109]. Demethoxycurcumin and bisdemethoxycurcumin suppress β-catenin transcription activated by Wnt3a-conditioned medium and inhibit the growth of various colon cancer cells [110]. Niclosamide and silibinin inhibit LRP6 expression and phosphorylation, block Wnt3a-induced β-catenin accumulation, and inhibit Wnt/β-catenin signaling in HEK293 cells, prostate cancer PC-3 and DU145 cells, and breast cancer MDA-MB-231 and T-47D cells [111, 112]. Verapamil improves Wnt3a-induced loss of proteoglycan in chondrogenically differentiated ATDC5 cells [113].

Furthermore, retinoic acid can reduce *Wnt3a* mRNA expression in NT2 cells. Wnt3a expression is down-regulated by β-estradiol in MCF-7 cells [49]. Deep-sea water treatment results in a concomitant decrease in mRNA levels of *MMP*-*9*, transforming growth factor-β (*TGF*-*β*), *Wnt5a*, and *Wnt3a* in human breast cancer MDA-MB-231 cells [114]. All-trans-retinoic acid down-regulates the expression of Wnt3a, Wnt8a, Wnt8b, Wnt10b, and Wn11 in NT2 cells [115]. Magnolol inhibits Wnt3a-induced β-catenin translocation and subsequent target gene expression in human embryonic kidney 293 cells [116].

## Conclusions

Wnt3a, an important member of the Wnt family, activates the canonical Wnt signaling pathway to exhibit its biological functions. It is mainly involved in embryonic development, neural development, cell differentiation, proliferation, and tumorigenesis. Overall, our understanding of Wnt3a is incomplete, and molecular mechanisms of its specific roles remain to be further studied in various diseases, including cancer.
